# Herring supports Northeast Pacific predators and fisheries: Insights from ecosystem modelling and management strategy evaluation

**DOI:** 10.1371/journal.pone.0196307

**Published:** 2018-07-06

**Authors:** Szymon Surma, Tony J. Pitcher, Rajeev Kumar, Divya Varkey, Evgeny A. Pakhomov, Mimi E. Lam

**Affiliations:** 1 University of British Columbia, Institute for the Oceans and Fisheries, Vancouver, British Columbia, Canada; 2 Centre for Fisheries Ecosystems Research, Fisheries and Marine Institute of Memorial University of Newfoundland, St. John’s, Newfoundland and Labrador, Canada; 3 Department of Earth, Ocean and Atmospheric Sciences, University of British Columbia, Vancouver, British Columbia, Canada; 4 Hakai Institute, Heriot Bay, British Columbia, Canada; 5 Centre for the Study of the Sciences and the Humanities, University of Bergen, Bergen, Norway; Swedish University of Agricultural Sciences and Swedish Institute for the Marine Environment, University of Gothenburg, SWEDEN

## Abstract

This paper analyzes the trophic role of Pacific herring, the potential consequences of its depletion, and the impacts of alternative herring fishing strategies on a Northeast Pacific food web in relation to precautionary, ecosystem-based management. We used an Ecopath with Ecosim ecosystem model parameterized for northern British Columbia (Canada), employing Ecosim to simulate ecosystem effects of herring stock collapse. The ecological impacts of various herring fishing strategies were investigated with a Management Strategy Evaluation algorithm within Ecosim, accounting for variability in climatic drivers and stock assessment errors. Ecosim results suggest that herring stock collapse would have cascading impacts on much of the pelagic food web. Management Strategy Evaluation results indicate that herring and their predators suffer moderate impacts from the existing British Columbia harvest control rule, although more precautionary management strategies could substantially reduce these impacts. The non-capture spawn-on-kelp fishery, traditionally practiced by many British Columbia and Alaska indigenous peoples, apparently has extremely limited ecological impacts. Our simulations also suggest that adopting a maximum sustainable yield management strategy in Northeast Pacific herring fisheries could generate strong, cascading food web effects. Furthermore, climate shifts, especially when combined with herring stock assessment errors, could strongly reduce the biomasses and resilience of herring and its predators. By clarifying the trophic role of Pacific herring, this study aims to facilitate precautionary fisheries management via evaluation of alternative fishing strategies, and thereby to inform policy tradeoffs among multiple ecological and socioeconomic factors.

## Introduction

Forage fish constitute an important energy conduit from zooplankton to higher predators [1–4]. Many aboriginal, recreational, subsistence and commercial fisheries also rely on forage fish, either directly or indirectly, i.e., via their support for piscivorous fish production [1, 2, 5]. Therefore, exploiting forage fish may reduce the ecosystem services they provide to charismatic and fished predators.

The importance of forage fish to predators and fisheries is particularly noticeable in temperate and upwelling ecosystems [1–5]. However, the occurrence and magnitude of yield tradeoffs between predator and forage fish fisheries vary greatly across ecosystems due to predator trophic ontogeny (juveniles of predatory fish species are largely planktivorous), as well as food web redundancy and complexity [6–9]. Furthermore, the predominant direction of control in trophic interactions between forage fish and their predators is often unclear [5, 8]. There are indications that many forage fish exert bottom-up control over their predators [1, 2, 5]. This effect appears to be common among central place foragers and mobile predators forming local feeding aggregations, e.g., most seabirds [4, 10] and many marine mammals, and less prevalent among predatory fish [7]. Conversely, several fish and seal species in the North Sea [5] and humpback whales (*Megaptera novaeangliae*) in the Northeast Pacific [11, 12] may exert moderate top-down control over forage fish, as has recently been predicted in a theoretical model [6].

Forage fish are characterized by an *r*-selected life history strategy, including stock-recruitment curves with a high steepness near the origin, high fecundities and natural mortality rates, short lifespans and low ages at maturity [2]. These traits, combined with the close link between forage fish abundance and oceanographic drivers, cause drastic population fluctuations [2] over interannual to multidecadal time scales [13, 14]. Such variability renders forage fish particularly vulnerable to stock collapses [7, 15], especially when stock biomass overestimates in less productive years promote unsustainable fishing mortality rates [2, 16]. This vulnerability to exploitation, combined with their importance to fished, charismatic and protected predators, has inspired global calls for precautionary and ecosystem-based forage fish management [1, 2]. Foremost among the distinguishing goals of ecosystem-based fisheries management (EBFM) is an explicit attempt to preserve the structure and function of the entire food web, including the needs of predators and protected species [17–19].

Pacific herring (*Clupea pallasii*) is a relatively large and long-lived forage fish inhabiting North Pacific neritic food webs from the Sea of Japan to the California Current [20]. Particularly during the spawning season [21], it is a significant prey item for numerous fishes [22, 23, 24], marine mammals [11, 25] and seabirds [26, 27], as well as the target of many commercial, recreational, and aboriginal fisheries [14, 28, 29]. Fisheries exploit either adult herring (mainly pre-spawning females for the roe market) or eggs laid on prepared kelp fronds (spawn-on-kelp, SOK) or Western hemlock (*Tsuga heterophylla*) branches [30]. Both of the latter are traditional foods for many coastal North Pacific indigenous peoples, including the Tlingit [30], Haida [31], and Heiltsuk [32].

**Fig 1:**
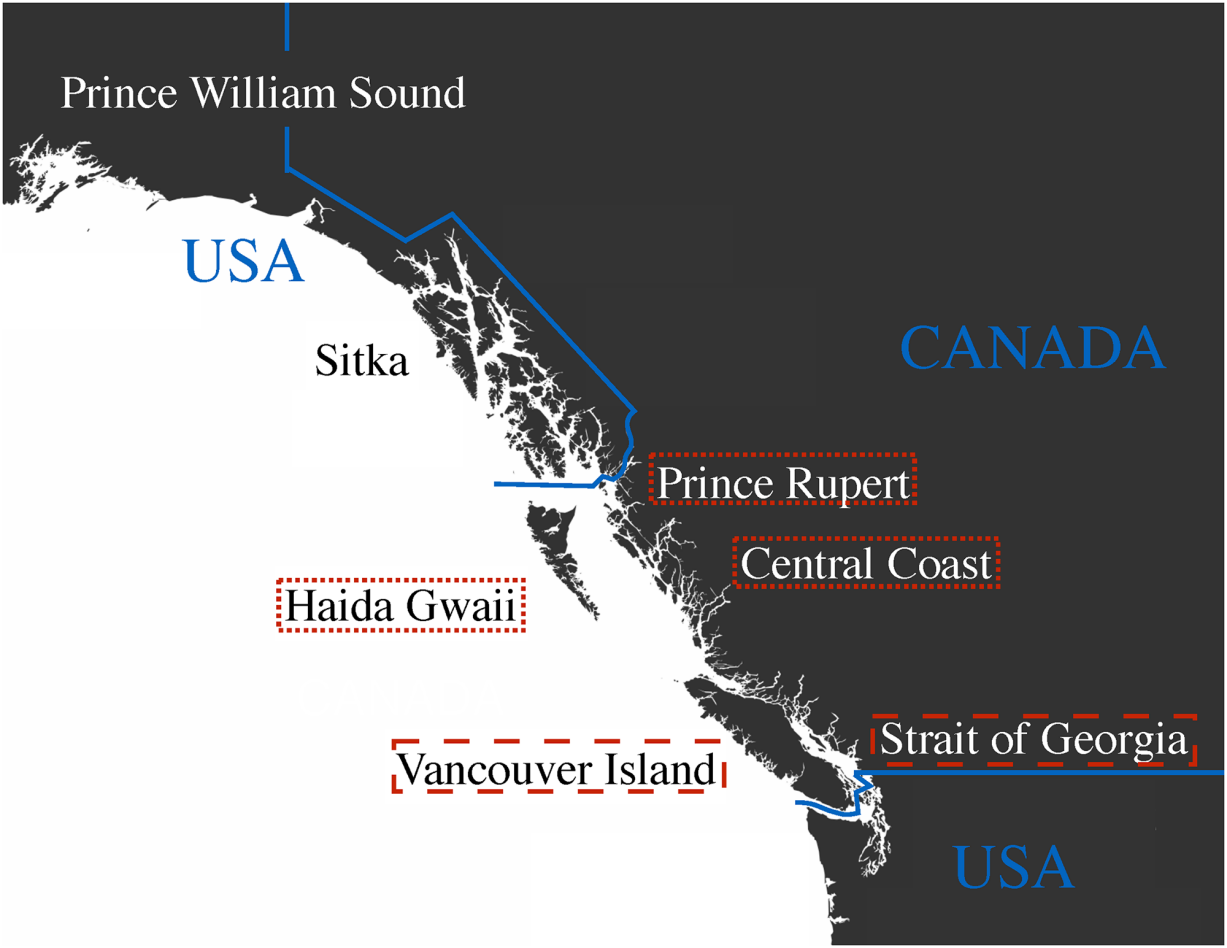
Major Northeast Pacific herring stocks. Red dotted borders around labels indicate British Columbia stocks included in this analysis, while red dashed borders refer to other stocks subject to the same management strategy. Alaskan stocks are managed using different strategies not investigated in this analysis.

**Fig 2:**
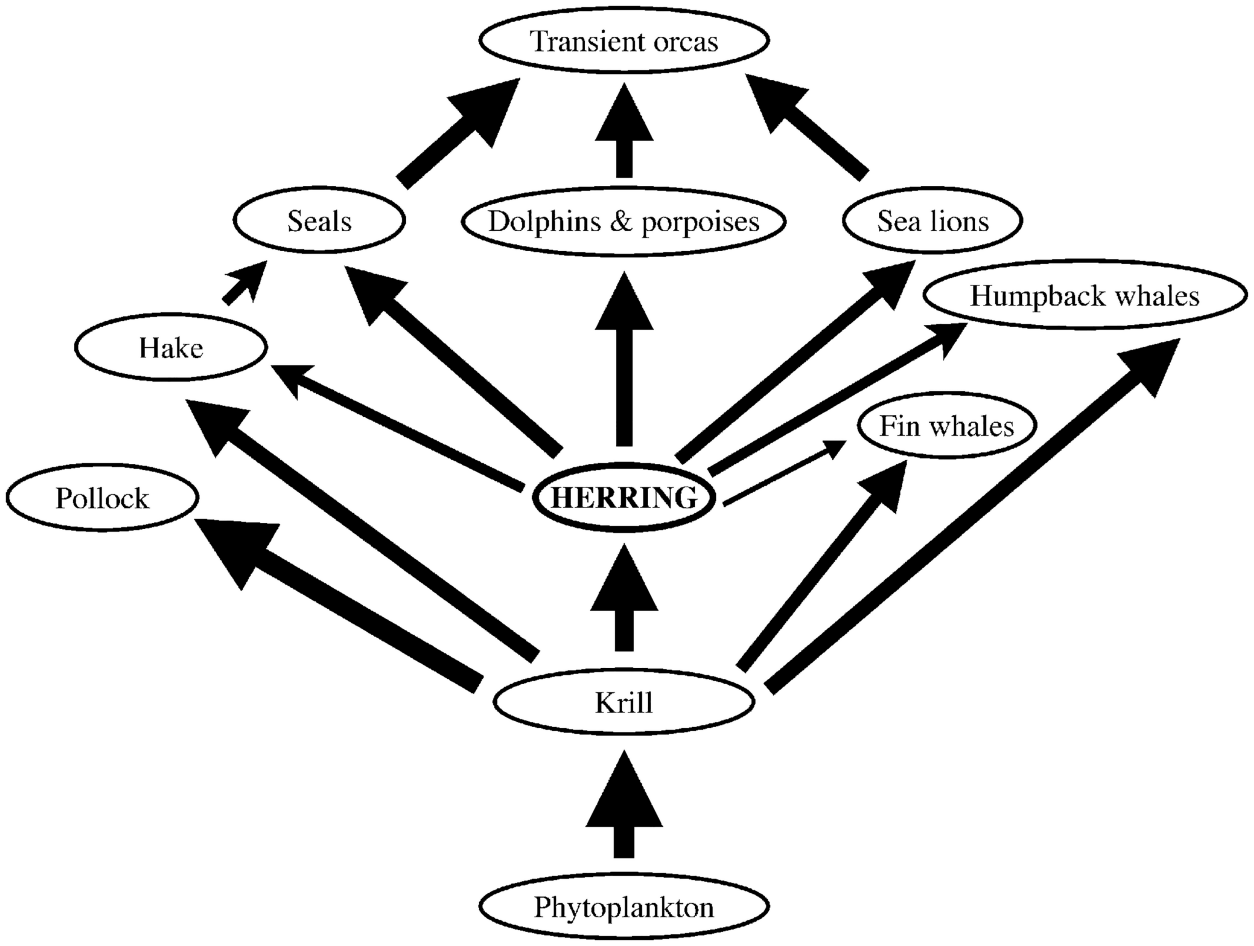
Simplified diagram showing the major trophic interactions involving herring in the modelled food web. Arrow thickness is proportional to biomass flux between functional groups.

In recent years, the poor status of many herring stocks in British Columbia and Alaska (Fig 1) has raised increasing concern among scientists [33–35], government agencies [14, 28] and aboriginal groups [30–32]. This has led to calls for more precautionary EBFM approaches, despite claims that current management strategies are sufficiently precautionary from a single-species perspective [33, 36, 37]. Current stock abundances are clearly below both estimated carrying capacities [38, 39] and prehistoric abundances inferred from zooarchaeological data [40]. Neither bottom-up nor top-down effects alone can satisfactorily explain this situation [12, 28, 34, 35]. In Prince William Sound, (Alaska, Fig 1), density-dependent effects and freshwater discharge [41], infectious disease [42] and the *Exxon Valdez* oil spill [43] may also have affected herring stock status. The magnitude of current and future effects of herring stock depression on food webs and predators is highly uncertain, and improved fisheries management requires in-depth knowledge of trophic interactions between Pacific herring and its predators (Fig 2).

This paper quantifies Pacific herring trophodynamics in northern British Columbia, notably Haida Gwaii (an archipelago in northern British Columbia and the ancestral home of the indigenous Haida people), focusing on the support that herring provides to its predators and the impacts of fishing pressure on its biomass and ecosystem role. Our study also comparatively evaluates the effects of various management strategies on local herring and predator biomasses as well as herring fisheries performance, accounting for both top-down and bottom-up processes. We hope this research may aid policymakers in formulating more precautionary and ecosystem-based herring management strategies that could support food web structure and predator populations as well as sustainable commercial and aboriginal fisheries.

## Methods

Explicit incorporation of fisheries into food webs makes ecosystem modelling in a framework such as Ecopath with Ecosim (EwE) a useful tool for examining ecosystem impacts of management strategies [2, 44], as well as potential precautionary reference points and EBFM approaches [2, 18, 45]. Ecosystem modelling places forage fish (e.g. Pacific herring) in the context of the entire food web, from their prey (zooplankton) to their predators (seabirds, marine mammals, larger fish) and fisheries [1, 2, 5, 8, 46]. In EwE, Ecopath creates, using the principle of mass balance, a static food web model that forms the basis for the calculation of various ecological metrics and dynamic ecosystem simulations in Ecosim [47]. The latter projects the Ecopath model forward to investigate effects of top-down and bottom-up forcing, as well as fishing pressure, on food web structure and function.

### Ecopath model

All analyses were conducted using the Ecopath model of northern British Columbia waters described in [22]. Three separate herring stocks, i.e., Haida Gwaii (HG), Prince Rupert (PRD) and Central Coast (CC), were covered together in this model. To better align our model outputs with those of the integrated statistical catch-at-age model (ISCAM) used for herring stock assessments in British Columbia [14], our model’s total (adult + juvenile) herring biomass was slightly reduced to place it within the 95% confidence interval of the ISCAM total biomass estimate. Biomasses of humpback whales, dolphins and porpoises, seals and sea lions were therefore decreased to the highest values which satisfied Ecopath’s basic principle of mass balance given the reduced herring biomass, while remaining compatible with field survey results from northern British Columbia [48, 49] and southeast Alaska [50]. The full parameters of this modified model are given in S2 and S3 Files.

Unlike many previous ecosystem models used to simulate effects of forage fish fisheries on predator populations [51], our model contains separate functional groups for two large forage fish (herring and eulachon *Thaleichthys pacificus*), with only several smaller species (Pacific sandlance *Ammodytes hexapterus*, capelin *Mallotus villosus* and small smelts) aggregated in a single “forage fish” group [22]. Furthermore, to account for the complex effects of trophic ontogeny and size-selective predation on forage fish trophodynamics [51, 52], we separately modelled two herring age/size classes (adult and juvenile), linked by a Beverton-Holt stock-recruitment relationship [22] using the Ecopath multi-stanza representation of age structure [47]. Most predators in our model consume more adult than juvenile herring biomass [22], which restricts the effect that size-selective predation on juveniles has on interactions between herring and its predators.

**Fig 3:**
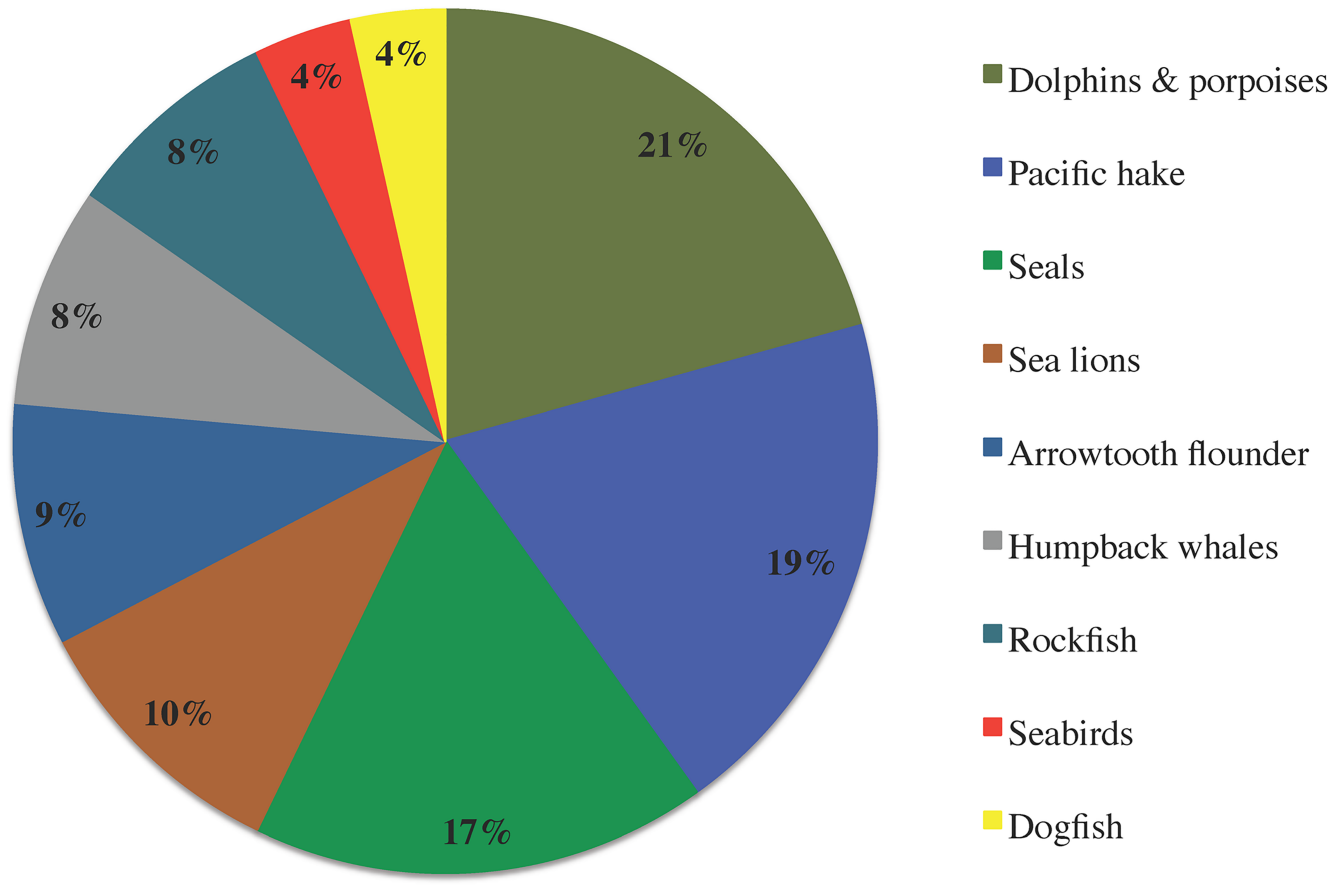
Proportions of total herring consumption by predators attributable to each predator in the Ecopath model.

**Fig 4:**
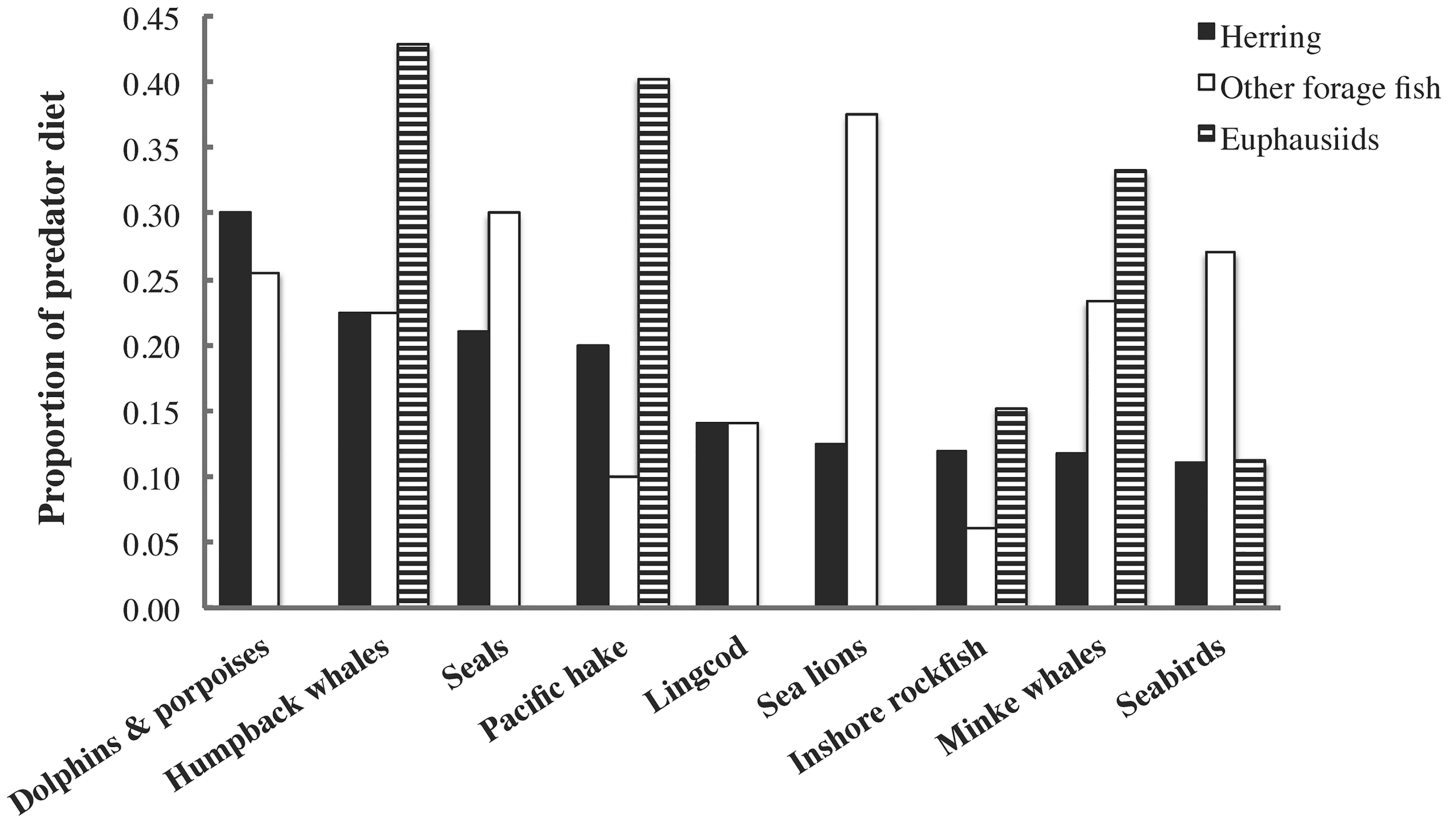
Proportions of herring, other forage fish (except eulachon) and euphausiids in selected predator diets from the Ecopath model.

The relative herring biomass flux to each predator, i.e., the proportion of total herring consumption by predators attributable to each predatory functional group, is shown in Fig 3. A preliminary estimate of the dependence of each predator on herring compared to other forage fish and euphausiids can be obtained from the model diet composition matrix (Fig 4). We hypothesized that predators with diets including ≥ 20% herring (i.e., dolphins and porpoises, humpback whales, seals, and Pacific hake *Merluccius productus*) would be particularly vulnerable to herring biomass changes, and used this as one criterion to explore ecosystem impacts of herring fisheries.

### Ecosim dynamic simulations

An Ecosim scenario with no herring fishery (*F* = 0) for 100 years was used as a baseline to investigate fishing impacts. To simulate the effects of herring stock collapse on functional group biomasses and food web structure, we ran an Ecosim scenario applying an extreme, constant fishing mortality rate (*F* = 2.4) to all herring fisheries for 100 years. *F* for all other fisheries remained at Ecopath baseline levels. To reproduce realistic patterns of potential ecosystem change, control for effects of top-down and bottom-up forcing and reproduce natural herring population fluctuations, as recommended in [46], we employed time series (S4 File) based on those introduced in [12]. Time series driving projected future whale recovery were deactivated in one of the two Ecosim runs per scenario to examine the effects of herring depletion on humpback, blue (*Balaenoptera musculus*), fin (*B*. *physalus*), sei (*B*. *borealis*) and sperm (*Physeter macrocephalus*) whale biomasses. Stochastic interannual variability in phytoplankton biomass was represented by Monte Carlo resampling of values for each simulated year from a time series stretching from 1950 to 2000 [53].

### EwE management strategy evaluation (MSE) simulations

EwE includes a management strategy evaluation (MSE) module designed to investigate effects of harvest-control-rule (HCR) types, defined by biomass limit (*B*_*lim*_) and target fishing mortality rate (*F*_*target*_) reference points, on target fish stocks and entire food webs. A classical MSE analysis [54, 55] involves a closed-loop simulation of both human (i.e., assessment and management) and ecological subsystems affecting management strategy performance. Its objective, not pursued in most stock assessment models [56], is to comparatively evaluate the performance of different strategies against a chosen set of quantitative metrics reflecting single-species, ecological and/or socioeconomic criteria.

Several studies have employed MSE simulations within food web models built in EwE [2], Atlantis [57], or OSMOSE [58] to evaluate potential EBFM strategies. While the EwE MSE module was not intended to constitute a full MSE of the kind recommended [18, 55] for addressing EBFM (as it lacks full assessment and management subsystem simulations), it contains the essential elements of all subsystems and the entire MSE methodology. Furthermore, its direct link to a food web model and ability to emulate stochastic stock assessment error render it a promising tool for EBFM analysis. For instance, this module was employed by the Lenfest Forage Fish Task Force [2] to search for precautionary EBFM strategies for forage fish fisheries in coastal food webs. The authors concluded that strategies based on low *F*_*target*_ and high *B*_*lim*_ combined with a hockey-stick HCR had lowest impacts on predator biomasses and thus were most precautionary. MSE has also been applied to British Columbia herring fisheries within a single-species framework, largely supporting the current management strategy [33, 36, 37]. In the latter [14], *B*_*lim*_ = 0.25*B*_*0*_, where *B*_*0*_ = unfished biomass. If stock biomass *B* < *B*_*lim*_, *F* = 0 (the fishery is closed). Typically if *B* ≥ *B*_*lim*_, *F*_*target*_ = 0.2, but management officials may recommend *F*_*target*_ = 0.1 in response to requests for greater precaution from scientists or aboriginal groups. In our EwE MSE simulations, *B*_*0*_ = total herring biomass produced by an Ecosim run in which *F* = 0 for 1000 years (2.7 t/km^2^).

The EwE MSE module allows users to specify whether each modelled fishery is managed using output (e.g., quotas) or input (e.g., limited entry) controls. All our scenarios used quotas to replicate current Northeast Pacific herring fisheries management, such that the modelled herring fishery each year is based on a simulated stock assessment which estimates biomass to set the quota. Stochastic stock assessment error for each target functional group (in this case, adult herring) was simulated within the EwE MSE by Monte Carlo resampling of biomass estimates from a normal distribution centered on the modelled biomass and characterized by a chosen coefficient of variation (CV). As a tradeoff between allowing realistic biomass uncertainties in stock assessments and avoiding software crashes, we set CV = 0.3 for adult herring and CV = 0 for all other groups. Each EwE MSE scenario run was repeated 100 times for this herring biomass resampling and lasted 100 simulated years.

**Fig 5:**
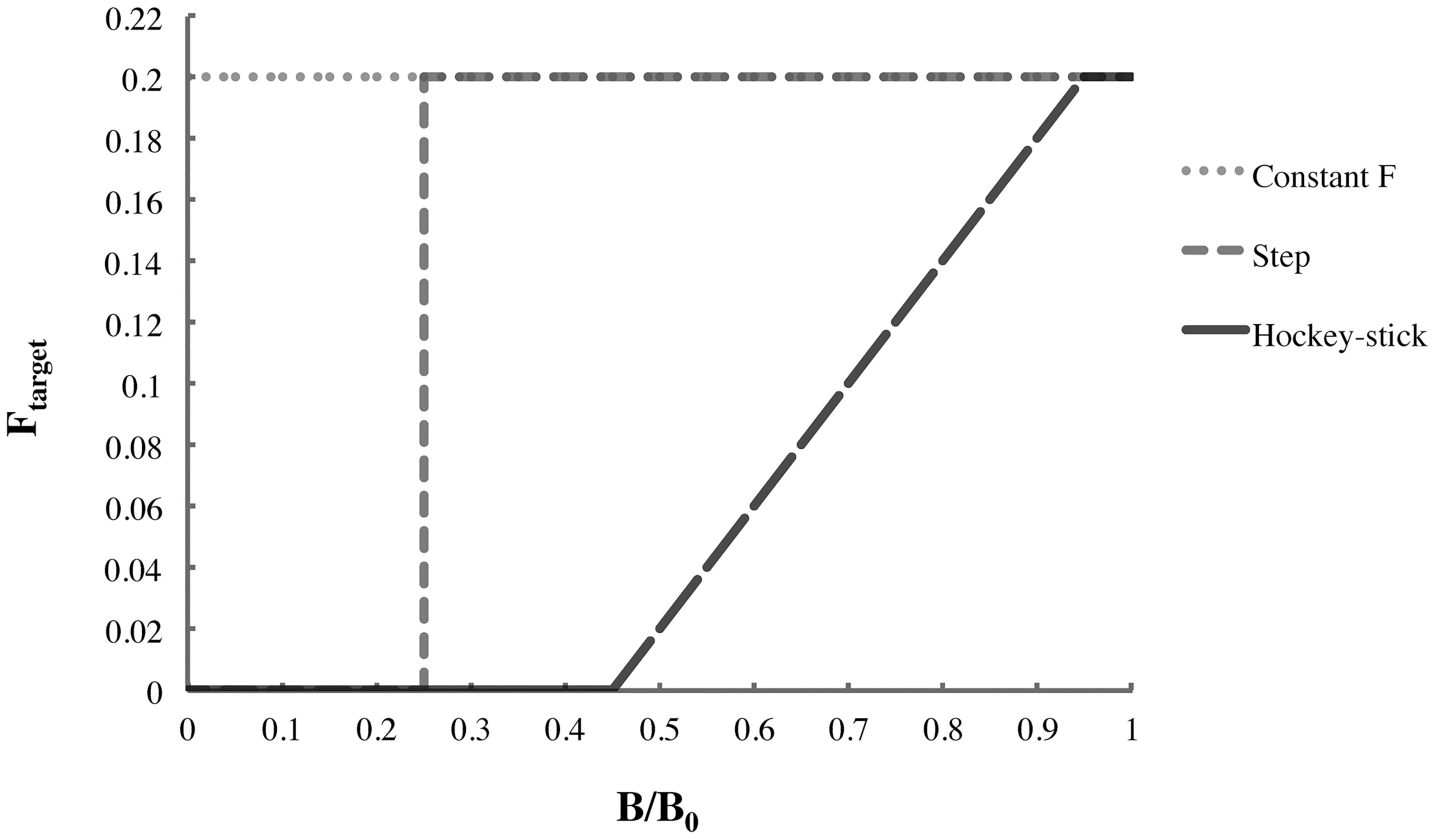
Constant *F*_*target*_, step and hockey-stick harvest-control rules for target fishing mortality rate *F*_*target*_ = 0.2.

**Fig 6:**
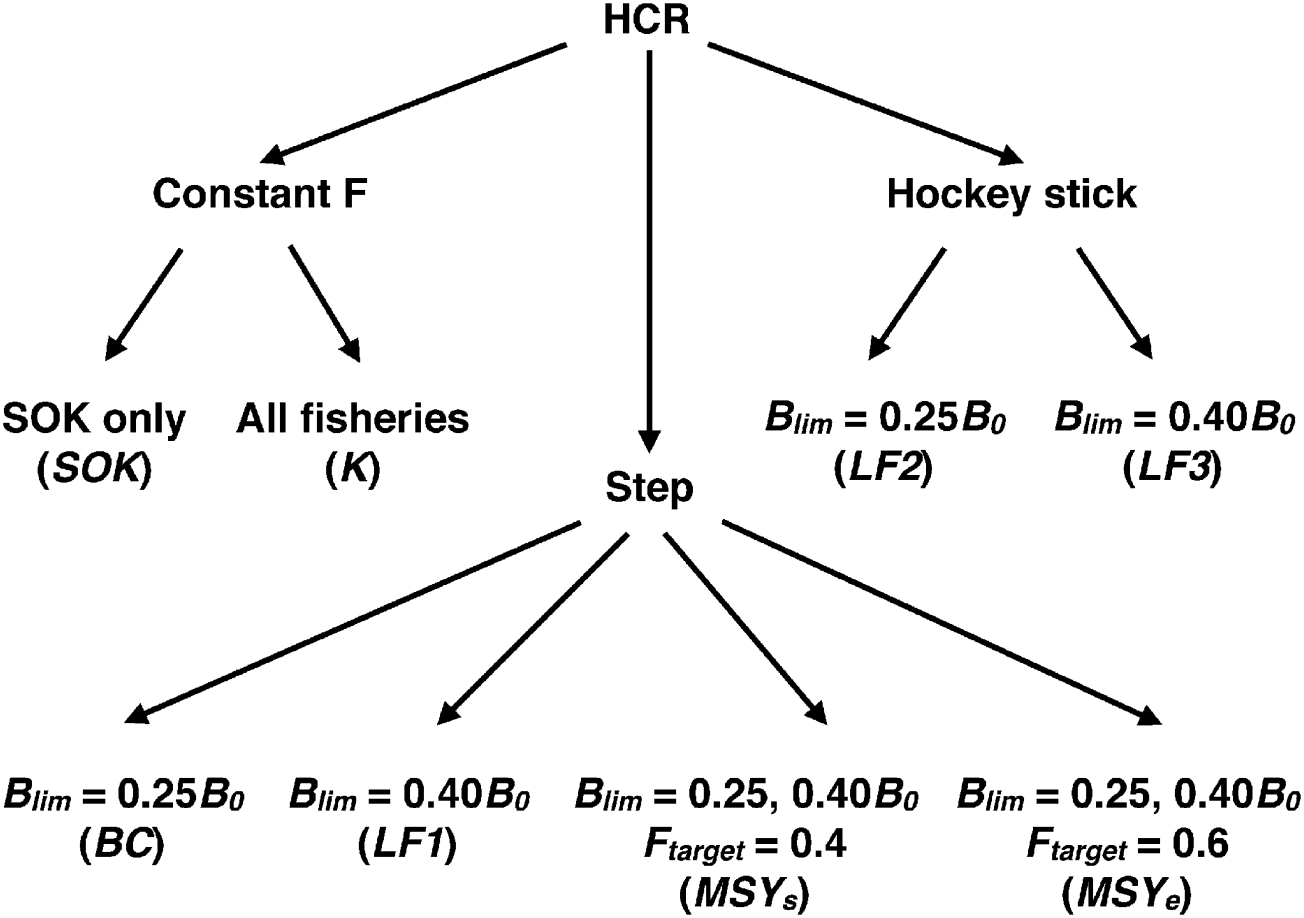
The management strategy evaluation scenarios classified by harvest-control-rule type, *F*_*target*_ and *B*_*lim*_.

The EwE MSE module permits researchers to investigate the ecological impacts of various management strategies, here defined by three HCR types (constant *F*_*target*_, step, and hockey-stick) and the values of three reference points for target fish stocks: *F*_*target*_, *B*_*lim*_ and *B*_*Ftarget*_, the biomass at which *F*_*target*_ is reached (Fig 5). All EwE MSE scenarios investigated in this study are shown in Fig 6 and Table 1, classified by their HCR types and *F*_*target*_, *B*_*lim*_ and *B*_*Ftarget*_ values. We compared constant *F*_*target*_ with both the step HCR currently used in British Columbia herring management [14] and the hockey-stick HCR found to be precautionary by the Lenfest Forage Fish Task Force [2]. The complex step + hockey-stick HCR [59] employed to manage herring fisheries in neighbouring southeast Alaska (Fig 1), cannot be simulated by the EwE MSE module.

**Table 1:** EwE MSE scenarios and their parameter values. Scenario codes are explained in the text.

Scenario	Fisheries	HCR	*B*_*lim*_	*B*_*Ftarget*_	*F*_*target*_	PP years
*Base*	None	constant	0	0	0	All
*SOK*	SOK	constant	0	0	0.01	All
*K*	All	constant	0	0	0.1, 0.2	All
*MSY*_*s*_	All	step	0.25*B*_*0*_ (*MSY*_*s1*_) 0.40*B*_*0*_ (*MSY*_*s2*_)	0.25*B*_*0*,_ (*MSY*_*s1*_) 0.40*B*_*0*_ (*MSY*_*s2*_)	0.4	All
*MSY*_*e*_	All	step	0.25*B*_*0*_ (*MSY*_*e1*_) 0.40*B*_*0*_ (*MSY*_*e2*_)	0.25*B*_*0*_ (*MSY*_*e2*_) 0.40*B*_*0*_ (*MSY*_*e2*_)	0.6	All
*LF1*	All	step	0.40*B*_*0*_	0.40*B*_*0*_	0.1, 0.2	All
*LF2*	All	hockey	0.25*B*_*0*_	0.95*B*_*0*_	0.1, 0.2	All
*LF3*	All	hockey	0.40*B*_*0*_	0.95*B*_*0*_	0.1, 0.2	All
*BC*	All	step	0.25*B*_*0*_	0.25*B*_*0*_	0.1, 0.2	All
*BC*_*0*.*75*_	All	step	0.25*B*_*0*_	0.25*B*_*0*_	0.2	No good
*BC*_*0*.*25*_	All	step	0.25*B*_*0*_	0.25*B*_*0*_	0.2	Only bad

EBFM requires setting precautionary values for reference points such as *B*_*lim*_ and *F*_*target*_. Proposed precautionary strategies include setting *F*_*target*_ < *F*_*MSY*_, the fishing mortality rate producing maximum sustainable yield [2, 3]. This is noteworthy given that according to both the reformed EU Common Fisheries Policy (CFP) of 2013 and the US Magnuson-Stevens Act (MSA), *F*_*target*_ = *F*_*MSY*_. However, individual European stocks (e.g. Norwegian spring-spawning herring) may be managed with precautionary hockey-stick HCRs in which *F*_*target*_ = *F*_*MSY*_ is the maximum possible *F* [60], while the US MSA prescribes *F*_*target*_ < *F*_*MSY*_ if ecological and/or socioeconomic concerns exist. Ecosystem modelling studies have also proposed setting forage fish *B*_*lim*_ ≥ 0.4*B*_*0*_ [2] or ≥ 0.25*B*_*0*_ [3], while a global analysis of field data suggested seabird populations alone require 0.33*B*_*0*_ to prevent declines [4].

**Fig 7:**
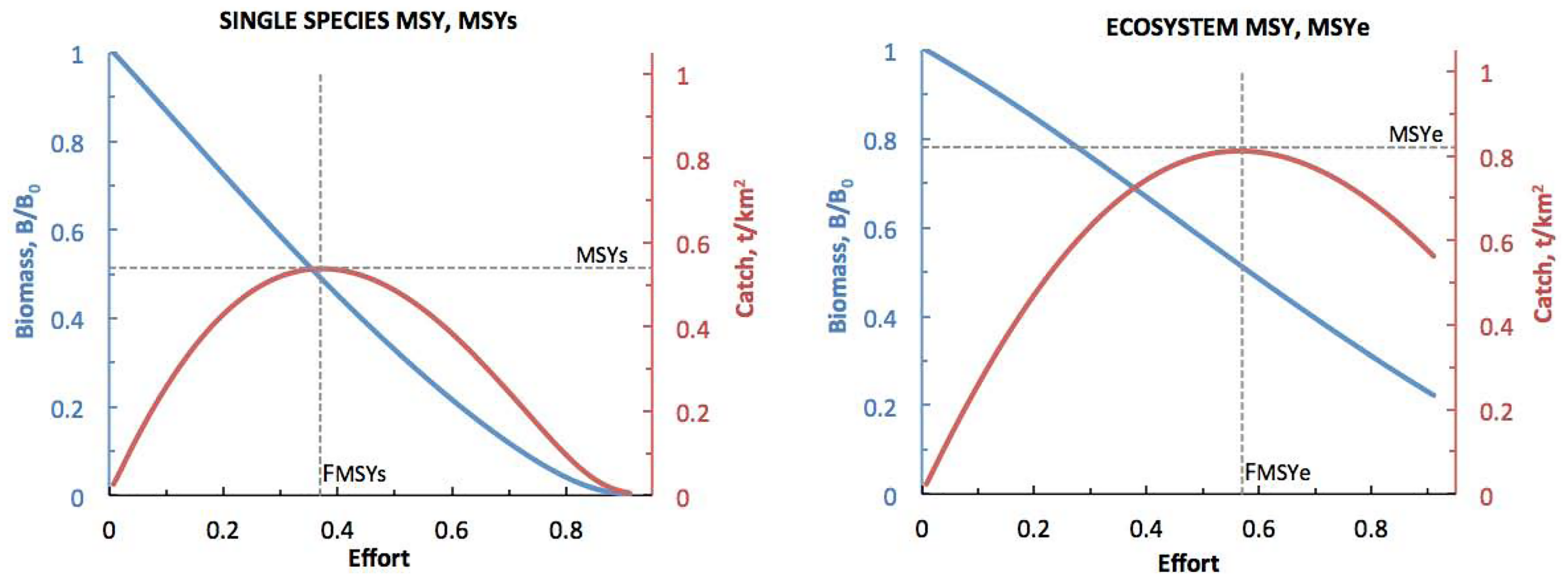
The two MSY estimates for adult herring derived from the EwE model. Single-species MSY_s_ and ecosystem-based MSY_e_ alike were obtained from the EwE MSY estimation tool.

We examined a set of EwE MSE scenarios based on constant *F*_*target*_, step or hockey-stick HCRs (Fig 6, Table 1). The constant *F*_*target*_ scenarios investigated were *K*, all commercial herring fisheries (i.e., roe, food and bait and spawn-on-kelp), and *SOK*, commercial spawn-on-kelp fishery only. Aboriginal SOK FSC fisheries were included in all scenarios. For *K*, *F*_*target*_ = 0.1 or 0.2 (minimum and maximum values used in British Columbia and Alaska), whereas for *SOK*, *F*_*target*_ = 0.01 (estimated maximum incidental mortality in SOK fisheries). The step scenarios were *BC* (*B*_*lim*_ = 0.25*B*_*0*_), the strategy used in British Columbia herring fisheries [14]; *LF1* (*B*_*lim*_ = 0.40*B*_*0*_), recommended as precautionary by the Lenfest Forage Fish Task Force [2]; and two maximum sustainable yield strategies, *MSY*_*s*_ and *MSY*_*e*_ (*B*_*lim*_ = 0.25*B*_*0*_ or 0.40*B*_*0*_ and *F*_*target*_ = *F*_*MSY*_). *F*_*MSY*_ ≈ 0.4 for single-species MSY (*MSY*_*s*_) and 0.6 for ecosystem-based MSY (*MSY*_*e*_), respectively [44], are derived from the EwE MSY estimation tool (Fig 7). For two hockey-stick scenarios, *LF2* and *LF3*, also suggested as precautionary by the Task Force [2], *B*_*lim*_ = 0.25*B*_*0*_ and 0.40*B*_*0*_, respectively, and *B*_*Ftarget*_ = 0.95*B*_*0*_. For the *BC* and *LF1-LF3* scenarios, *F*_*target*_ = 0.1 or 0.2, as in British Columbia and Alaska herring fisheries.

To comparatively evaluate management strategy performance and resulting ecosystem states, we employed several metrics and indicators, including functional group biomasses of herring and its predators, prey and competitors; mean and maximum herring catches; probabilities of herring fishery closure (*F* = 0) and stock collapse (*B* < 0.05*B*_*0*_); and the mean trophic level (*MTL*) and Shannon-Wiener *H’* biodiversity index of the food web (functional groups replaced species in *MTL* and *H’* calculations).

These metrics were computed from functional group biomass and herring catch distributions generated by the EwE MSE module. Distribution grand means were evaluated for each management strategy relative to a baseline scenario (*Base*, constant herring *F*_*target*_ = 0) by averaging over 100 Monte Carlo runs, each lasting 100 simulated years. Grand means minus 0.675 standard deviations (μ—0.675σ) were used to express risks associated with each scenario, i.e., the potential ecosystem impacts of low primary productivity combined with herring stock assessment error. Confidence intervals of twice the standard error (± 2SE) were employed to search for differences in group biomasses among scenarios as well as between the baseline scenario and each management strategy.

Time series driving primary productivity and whale recovery in Ecosim were used identically in all EwE MSE simulations described above. However, to investigate the potential performance of the *BC* management strategy under climate change or oceanographic regime shifts, two additional scenarios with associated phytoplankton biomass time series (*BC*_*0*.*75*_ and *BC*_*0*.*25*_) were prepared. *BC*_*0*.*75*_ only resampled values from below the third quartile of the historical [53] phytoplankton data distribution (“no good productivity years”), while *BC*_*0*.*25*_ only included values from below the first quartile (“only bad productivity years”).

### Model and parameter uncertainty

While EwE MSE simulations incorporate uncertainty by representing stock assessment error and interannual variability in primary productivity through Monte Carlo resampling, our analyses, as with all complex simulations, suffer from both model and parameter uncertainty.

The Ecopath model represents the relationship between functional group biomass and non-predation-related natural mortality linearly. For groups characterized by high density-dependent mortality, a quadratic representation may better capture control mechanisms in predator-prey interactions and propagation of bottom-up effects through the food web [61], but the requisite empirical data are difficult to obtain. The current model also does not represent non-consumptive, fear-mediated [62] top-down effects of top predators (e.g., large demersal sharks) on mesopredators (e.g., seals), with cascading positive effects on prey (e.g., herring). Such effects could be modelled using trophic mediation functions; however, the quantitative data needed for an accurate representation are not yet available. The lack of explicit age structure for most predatory fish (except Pacific halibut *Hippoglossus stenolepis*) in the present model precludes examining the effects of their trophic ontogeny on herring ecology [9, 51, 52], but our moderately detailed model should be sufficient to meet the goals of this study.

Ecosim assumes stock-recruitment relationships for all forage fish, which has been questioned [51]. However, the authors of this study admit that in the absence of such relationships, predators could still be affected by declines in forage fish standing stocks due to fishing. Another recent study [52] suggests that an absence of stock-recruitment relationships in predatory fish (also assumed by Ecosim) could buffer them against forage fish depletion, but its authors concede that predator-prey recruitment covariation due to common bottom-up drivers in shared nursery areas could counteract this effect.

Uncertainty in base Ecopath parameter (*B*, *P/B* and *Q/B*) values and the model diet composition matrix (S1 File) may affect simulation results, but could be remedied in future studies by Monte Carlo resampling of parameter values. Low functional group resolution (particularly for seabirds and zooplankton) in the current model may obscure important predator-prey interactions (S1 File), but will be enhanced in future model versions [22]. Sensitivity analyses (S1 File) showed that Ecosim and EwE MSE results are quite robust to uncertainty in Ecosim vulnerability parameters.

## Results

### Ecosim dynamic simulations

**Fig 8:**
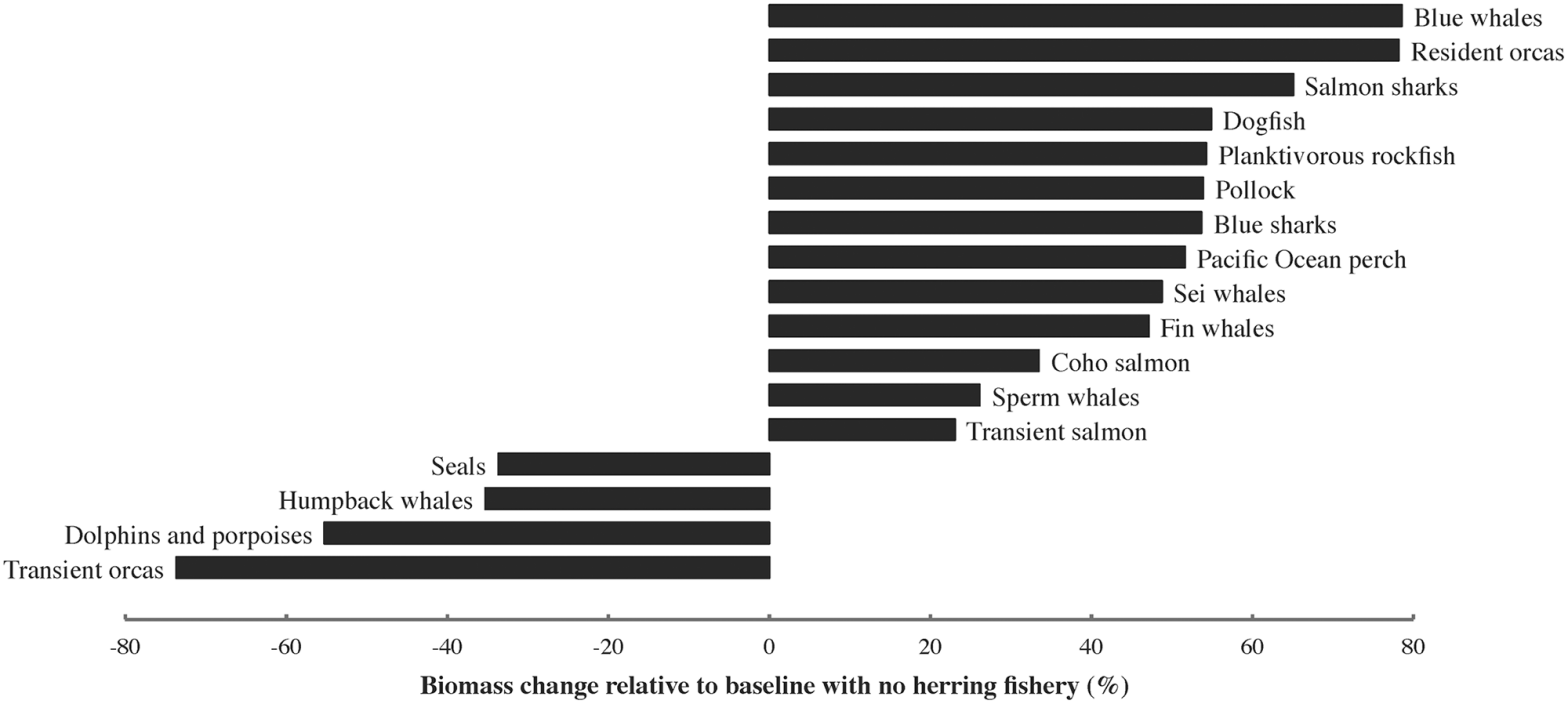
Functional group biomass changes (> 20% of baseline) in the Ecosim herring stock collapse simulation.

The food web responded dramatically to the modelled collapse (95% depletion) of herring (Fig 8, S5 File). As predicted, strong declines (≥ 25% baseline values) were registered for most functional groups whose diets comprise ≥ 20% herring, i.e., dolphins and porpoises, humpback whales and seals (Fig 3) as well as top predators (transient orcas *Orcinus orca*). By contrast, predominantly planktivorous mammals (blue, fin and sei whales) and fish (walleye pollock *Gadus chalcogrammus*, Pacific Ocean perch *Sebastes alutus*, other planktivorous rockfish *Sebastes* spp.) increased in biomass due to herring collapse and consequent reduced competition for their prey. This in turn raised the biomasses of several pelagic predators (sperm whales, resident orcas, Pacific dogfish *Squalus suckleyi*, salmon sharks *Lamna ditropis* and blue sharks *Prionace glauca*). Biomass changes exceeding 25% of baseline values impacted 33% of non-herring functional groups, including 75% of marine mammals, 60% of elasmobranchs and 60% of pelagic groups.

### EwE management strategy evaluation (MSE) simulations

**Fig 9:**
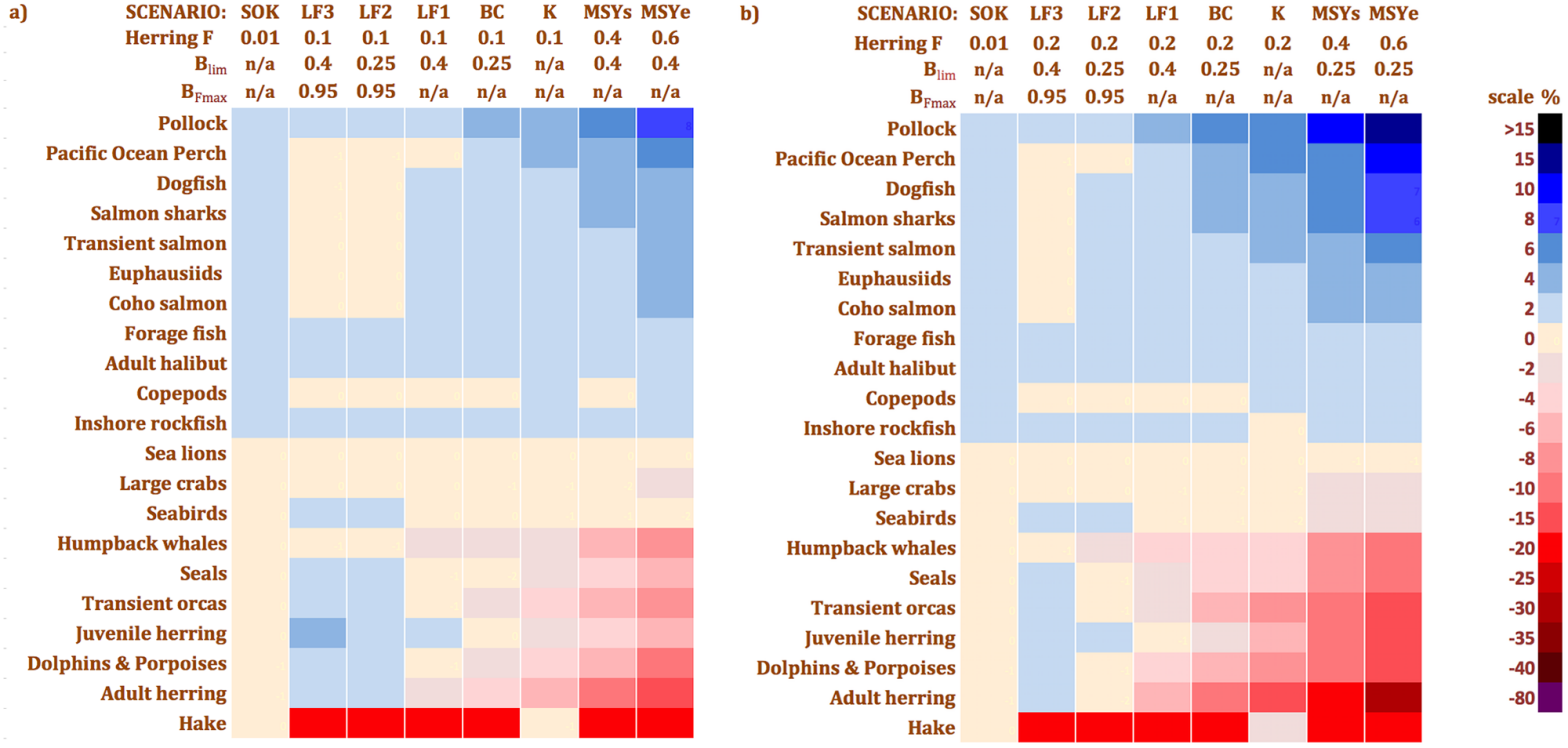
Grand mean functional group biomass changes by EwE MSE scenario. a) *F*_*target*_ = 0.1 except *SOK* (0.01) and the *MSY*_*s*_ and *MSY*_*e*_ scenarios (0.4 and 0.6, respectively, at the higher *B*_*lim*_) b) *F*_*target*_ = 0.2 except *SOK* (0.01) and the *MSY*_*s*_ and *MSY*_*e*_ scenarios (0.4 and 0.6, respectively, at the lower *B*_*lim*_).

Results of the EwE MSE simulation scenarios (Fig 6, Table 1) show that *F*_*target*_, *B*_*lim*_ and HCR type combine to determine herring management strategy effects on functional group biomasses (Fig 9; S1 and S6 Files). Stronger ecosystem impacts were associated with higher *F*_*target*_, lower *B*_*lim*_ and constant *F*_*target*_ ≥ step > hockey-stick HCRs. The low (constant) *F*_*target*_ = 0.01 of the *SOK* strategy produced minimal ecosystem impacts compared with the higher (constant) *F*_*target*_ = 0.1 or 0.2 of the *K* strategy, which in turn had much weaker impacts than the two (step) *F*_*MSY*_ scenarios (*F*_*MSYs*_ ≈ 0.4 and *F*_*MSYe*_ ≈ 0.6). Lower *B*_*lim*_ (0.25*B*_0_ vs. 0.40*B*_*0*_) produced stronger ecosystem impacts (*BC* vs. *LF1*, *LF2* vs. *LF3*) which were exacerbated by higher *F*_*target*_ (0.2 vs. 0.1), although the effects of *B*_*lim*_ alone were weaker than those of *F*_*target*_. Comparing HCR types at identical *F*_*target*_, *K* (constant *F*_*target*_) was only slightly outperformed by *BC* (step, *B*_*lim*_ = 0.25*B*_0_), which was in turn bettered by *LF2* (hockey-stick, *B*_*lim*_ = 0.25*B*_0_ and *B*_*Ftarget*_ = 0.95*B*_0,_).

In response to herring management strategies with 0.01 < *F*_*target*_ < *F*_*MSY*_ (i.e., 0.1 and 0.2), biomasses of 11% of functional groups (four marine mammals and two teleosts, including adult herring) changed noticeably relative to the baseline scenario (herring *F* = 0). Biomasses of herring, hake, humpback whales, dolphins and porpoises, and transient orcas decreased, while that of sei whales increased. Regardless of HCR type and *B*_*lim*_, biomass changes at *F*_*target*_ < *F*_*MSY*_ were quite modest in magnitude (< 10% for all groups except herring and hake), and did not constitute a major reorganization of food web structure (Fig 9; S1 File). For the two *MSY* scenarios, however (*F*_*MSYs*_ ≈ 0.4 and *F*_*MSYe*_ ≈ 0.6, step HCR, *B*_*lim*_ = 0.25*B*_0_ or 0.40*B*_0_), ecosystem impacts were stronger, with more intense biomass changes and more affected groups. An additional 15% of functional groups (five mammals, one shark and two teleosts) were noticeably affected. Seal and juvenile herring biomasses decreased relative to the baseline, while those of blue, fin and sperm whales, walleye pollock and blue sharks increased. No scenario caused noticeable biomass changes in sea lions, Pacific salmon (*Oncorhynchus* spp.), rockfish, non-herring forage fish or any group below the trophic level of herring (3.14).

**Fig 10:**
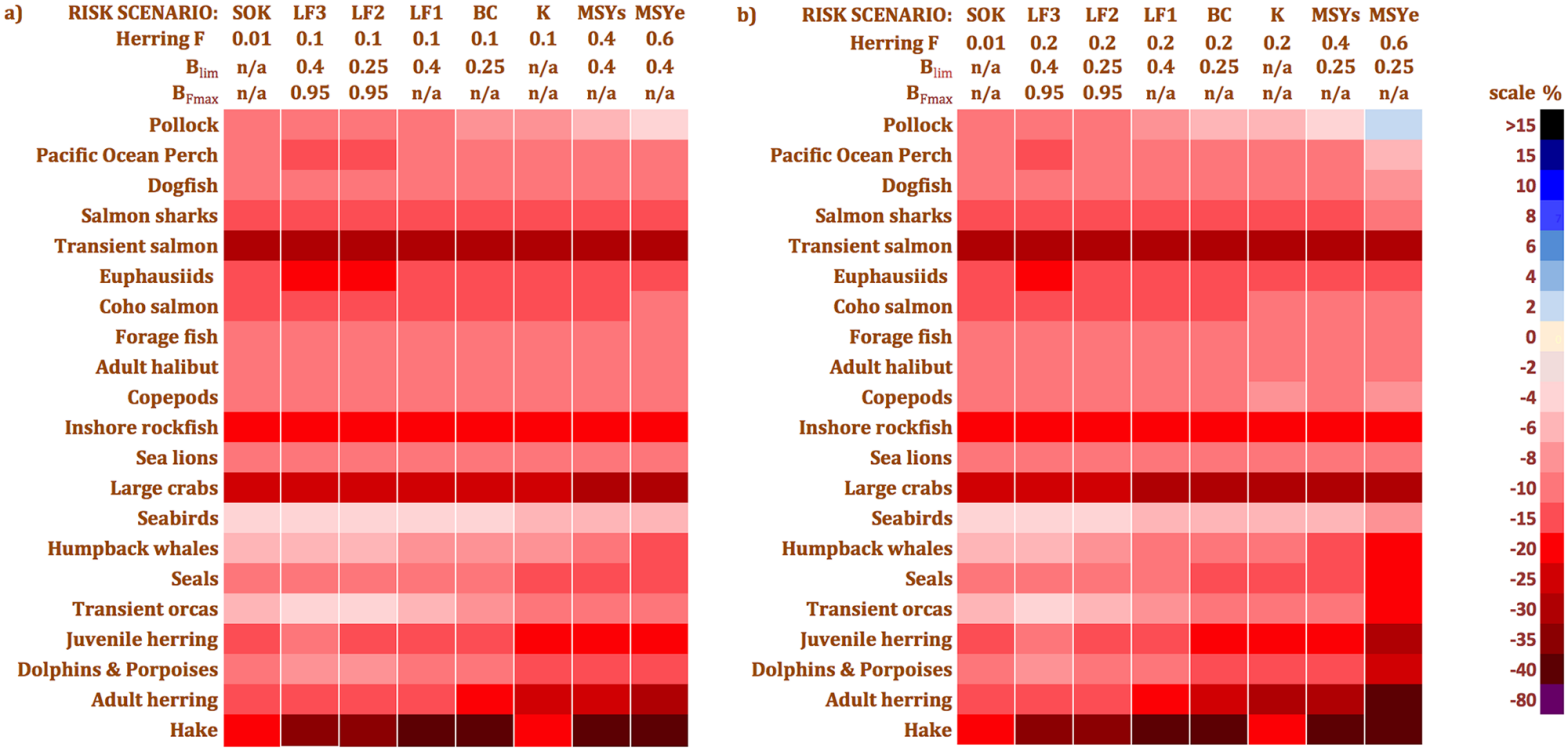
Grand mean minus 0.675 standard deviations (μ—0.675σ) functional group biomass changes showing the risks associated with EwE MSE scenarios. a) *F*_*target*_ = 0.1 except *SOK* (0.01) and the *MSY*_*s*_ and *MSY*_*e*_ scenarios (0.4 and 0.6, respectively, at the higher *B*_*lim*_) b) *F*_*target*_ = 0.2 except *SOK* (0.01) and the *MSY*_*s*_ and *MSY*_*e*_ scenarios (0.4 and 0.6, respectively, at the lower *B*_*lim*_).

**Fig 11:**
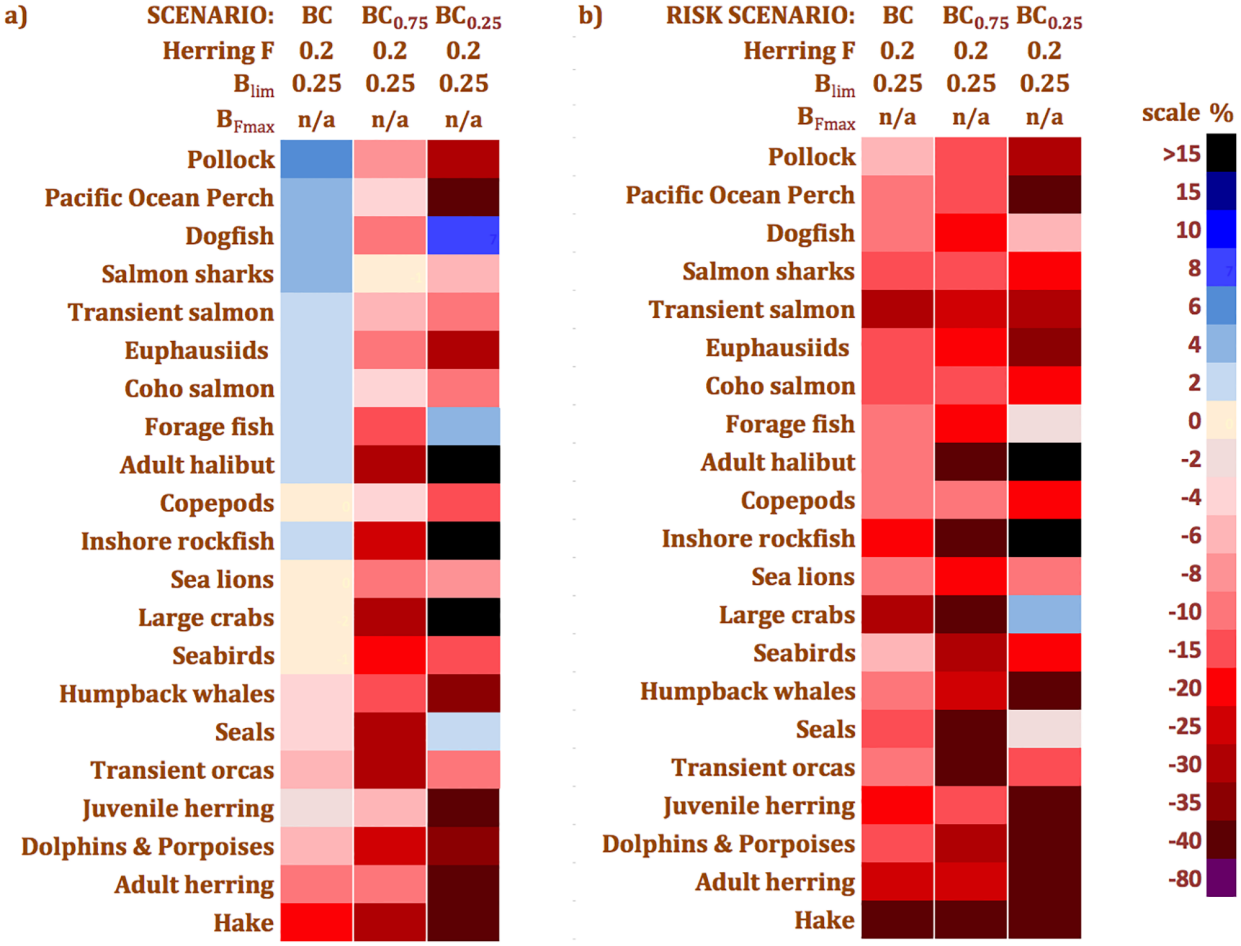
a) grand mean and b) grand mean minus 0.675 standard deviations (μ—0.675σ) group biomass changes for *BC* climate EwE MSE scenarios. *BC*_*0*.*75*_ simulates “no good productivity years” while *BC*_*0*.*25*_ represents “only bad years”.

However, when one examines herring management strategies in terms of risk, using not the grand mean but the μ -0.675σ functional group biomass changes for each scenario, the potential for notable ecosystem impacts under all strategies is revealed. The strong, negative biomass changes for many functional groups (Fig 10) display the risks of the herring management strategies due to stock assessment error and interannual fluctuations in phytoplankton biomass. These risks are in stark contrast to the rather mild ecosystem impacts seen in the grand means (Fig 9). Furthermore, simulated climate change / regime shift effects on the *BC* strategy based on randomized phytoplankton biomass time series derived from “only bad productivity years” (*BC*_*0*.*25*_) and “no good productivity years” (*BC*_*0*.*75*_) drastically reduced most functional group biomasses (Fig 11).

**Fig 12:**
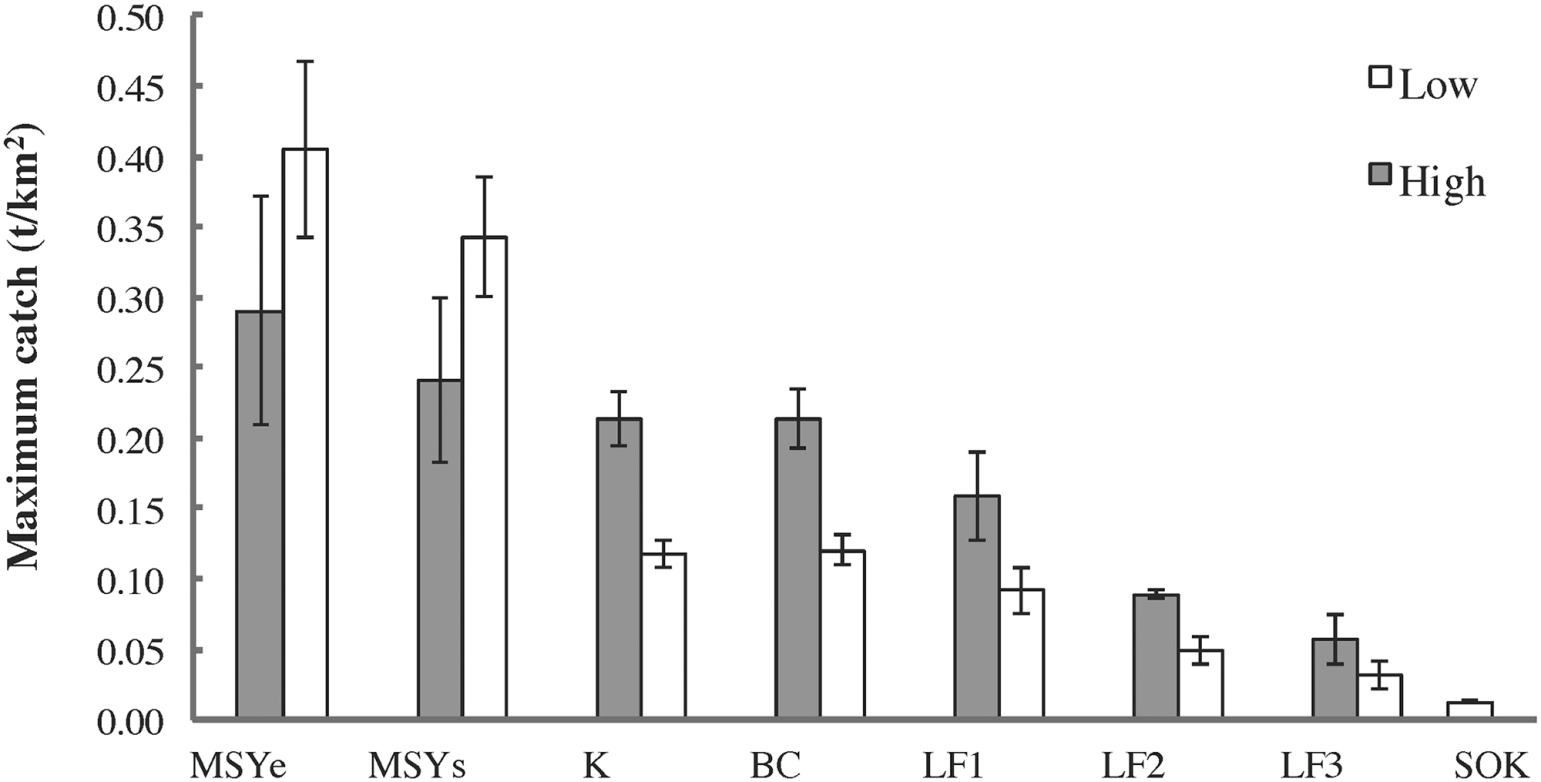
Grand mean herring catches by EwE MSE scenario. High and low: *B*_*lim*_ for *MSY*, *F*_*target*_ otherwise). Error bars indicate ±2 SE.

**Fig 13:**
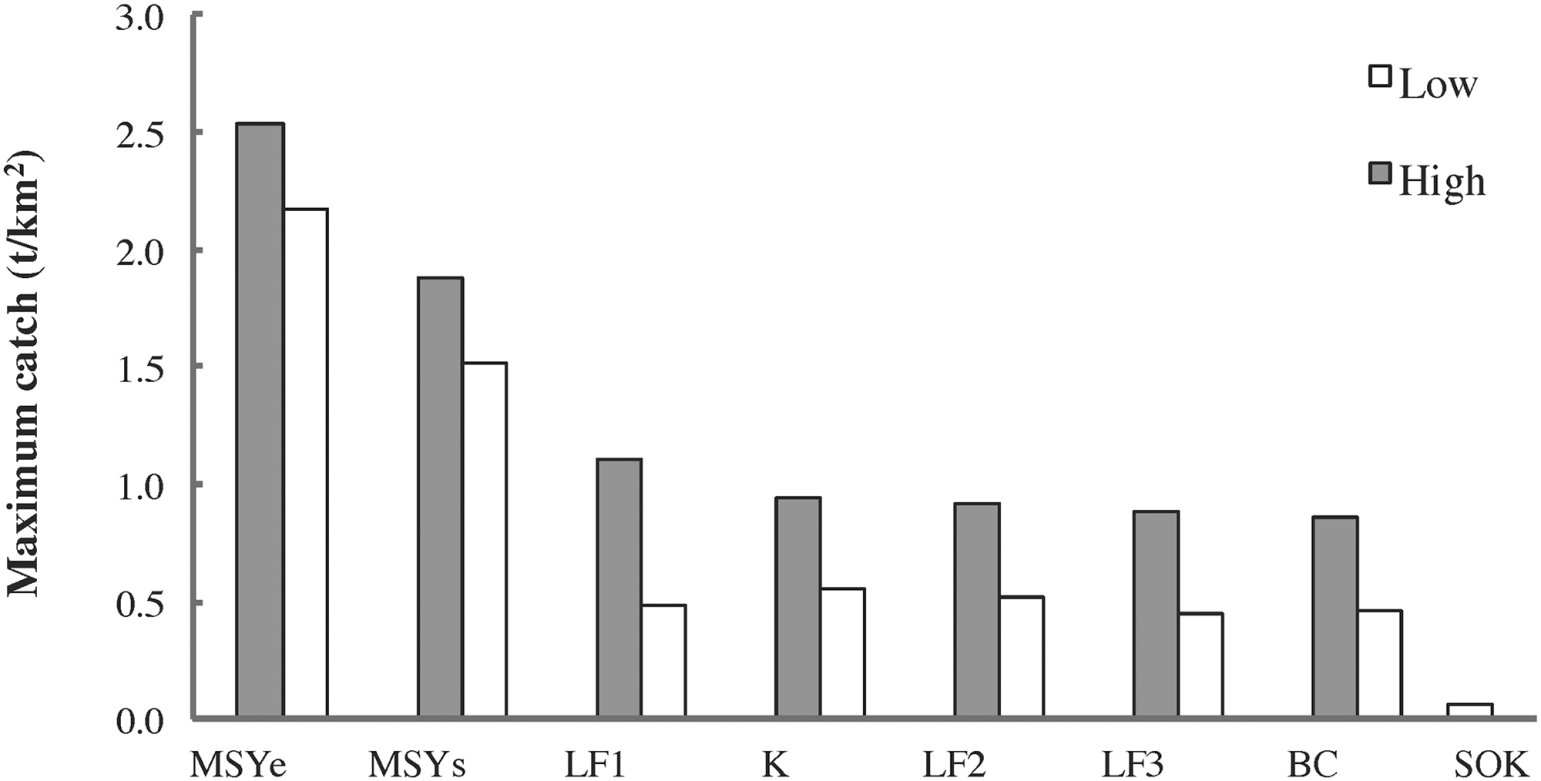
Maximum herring catches by EwE MSE scenario. High and low: *B*_*lim*_ for *MSY*, *F*_*target*_ otherwise.

**Fig 14:**
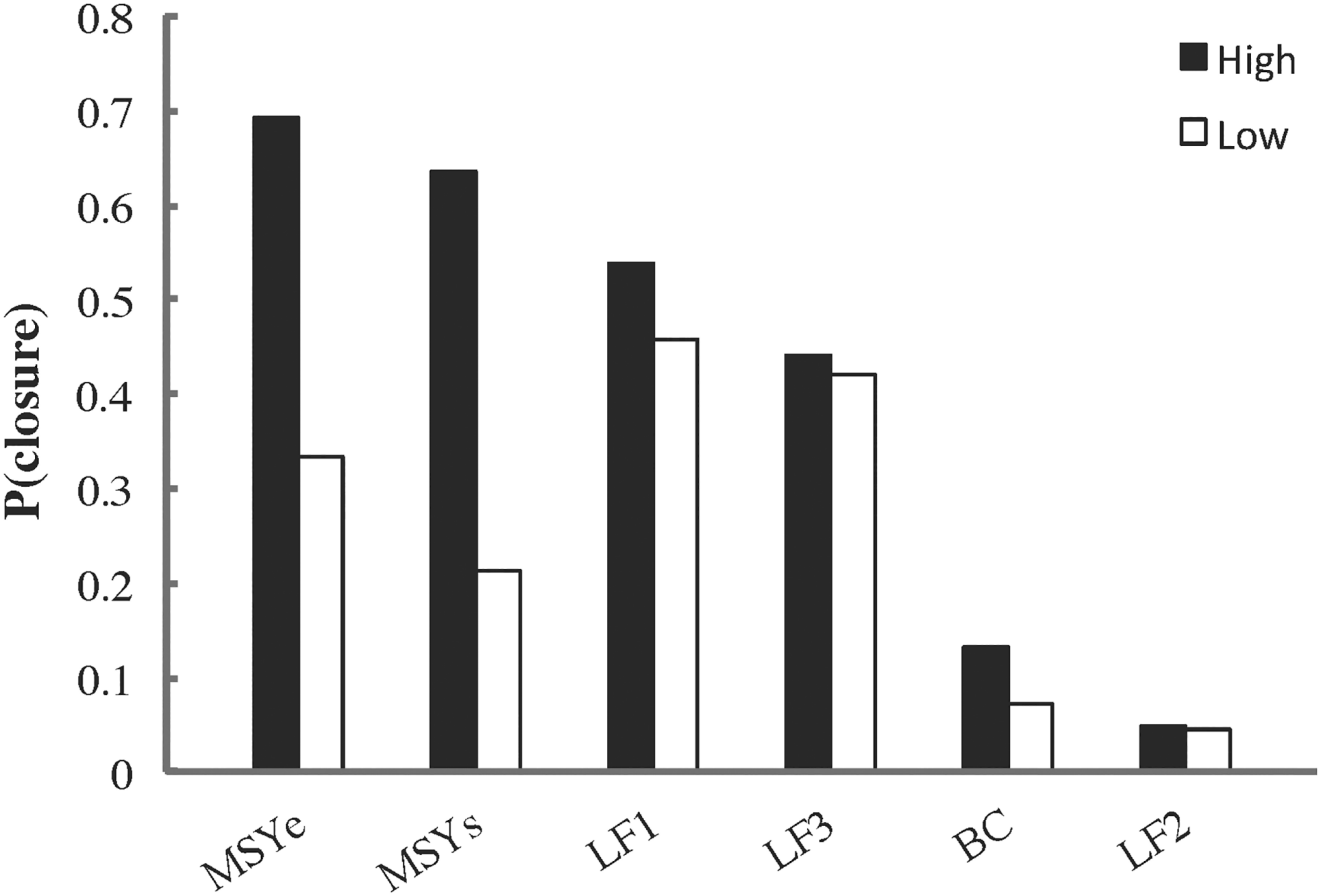
Probability of herring fishery closure by scenario. High and low: *B*_*lim*_ for *MSY*, *F*_*target*_ otherwise). For the two constant *F*_*target*_ scenarios (*SOK* and *K*), the fishery is always open (*B*_*lim*_ = 0).

Grand mean herring catches were greatest under management strategies incorporating high *F*_*target*_ and zero (constant *F*_*target*_; *K*) or low (step; *BC*) *B*_*lim*_ (Fig 12). Maximum herring catches were largely determined by *F*_*target*_, being highest and lowest under *MSY* and *SOK* scenarios, respectively (Fig 13). Probability of herring fishery closure was highest for both *MSY* scenarios at the higher *B*_*lim*_, followed by *LF1*, a step HCR with high *F*_*target*_ and *B*_*lim*_ (Fig 14). The probability of herring stock collapse (*B* < 0.05*B*_*0*_) was zero for all *F*_*target*_ < *F*_*MSY*_. All herring management strategies had negligible effects on the *MTL* (≤ 5%) and *H’* biodiversity index (≤ 1%), indicating little structural reorganization of the food web.

## Discussion

### Ecopath model

Herring is an important prey item for numerous predators, particularly marine mammals, within the Northeast Pacific food web (Figs 2–4), though its ecosystem role may not be as crucial as that of sardine and anchovy in upwelling ecosystems [1, 46, 63]. However, a recent study based on a set of energy-balanced Ecopath models suggests that Pacific herring may be more important as prey than mass-balanced models indicated [64], corroborating several studies [65, 66] which place adult herring among the most energy-rich forage fish in the subarctic North Pacific.

### Ecosim dynamic simulations

Results of the simulated herring stock collapse (Fig 8) suggest that herring is an important mid-trophic-level node in the Northeast Pacific food web (Fig 2). Mammalian predators whose diets contain ≥ 20% herring (humpback whales, dolphins and porpoises, seals; Fig 4) declined strongly, reducing the biomass of mammal-eating transient orcas. By contrast, many largely planktivorous fish and baleen whales benefited from reduced competition with herring for shared zooplankton prey, triggering increases in predators of planktivorous fish (dogfish, sperm whales, resident orcas, blue and salmon sharks). Effects of herring collapse in the Northeast Pacific would likely cascade through much of the food web to yield an ecosystem composition similar to that currently present in western Alaska, where forage fish and their pinniped predators have declined while walleye pollock have flourished following an oceanic regime shift [67]. Sensitivity analyses (S1 File) indicate that these Ecosim results are quite robust to model structure and parameter uncertainties.

### EwE management strategy evaluation (MSE) simulations

#### Role of herring in the food web

Many of the simulated herring management strategies noticeably reduced the biomasses of herring-eating mammals and the top predators reliant on those groups. Simultaneously, these strategies raised herring competitor biomasses (Fig 9). Forage fish trophic interactions, i.e., support for predators and competition with planktivores, are responsible for these effects, particularly the stronger bottom-up effects of forage fish fisheries on marine mammals [1, 2, 5, 8] relative to piscivorous fish [7]. While EwE typically predicts stronger impacts of forage fish fisheries on predators than does Atlantis due to more explicit age structure in the latter [52], our multi-stanza (age-structured) representations of herring and halibut at least partially mitigated this effect. Trophic ontogeny is much more pronounced among large fish than in marine mammals, making the interactions between herring and the latter less sensitive to this model uncertainty. The EwE MSE simulation results indicate that herring plays an important role in the Northeast Pacific food web as both prey to predators (Fig 3) and competitor to planktivores.

However, the modest herring fishing effects observed when *F*_*target*_ < *F*_*MSY*_ suggest that most management strategies examined here (Fig 6), including *BC*, will not substantially reorganize Northeast Pacific food webs, in contrast to forage fish fisheries in many upwelling ecosystems [2, 3]. This conclusion is supported by the lack of noticeable herring fishing effects on the *MTL* and Shannon-Wiener *H’* values of the modelled food web. This may be due to higher ecological redundancy in the Northeast Pacific forage fish guild relative to tropical upwelling systems [22, 63], as well as the diverse and flexible diets of most herring predators in the modelled ecosystem (Fig 4), as observed for forage fish predators in coastal waters throughout the continental USA [51].

Furthermore, for relatively omnivorous predators (e.g., fin whales, sea lions and seabirds), almost no net biomass changes were observed at *F*_*target*_ < *F*_*MSY*_, as herring fishing effects were balanced by increases in other forage fish or zooplankton competing with herring or consumed by herring, respectively. This complex relationship between herring, other forage fish and zooplankton may also explain herring fisheries effects on the highly omnivorous Pacific hake. Herring fishing effects on seabirds, however, may not be accurately quantified by our model due to the limited resolution of our seabird representation, which we are improving (S1 File). Previous studies [1–4] addressed the importance to seabirds of all forage fish rather than a single species (e.g., Pacific herring), hence their findings and ours are not directly comparable. In addition, multiple studies [10, 51, 68, 69] have found that local rather than total forage fish biomass may be particularly important to seabirds and other central place foragers. Such small-scale spatial effects cannot be simulated in the EwE MSE module, but may be investigated in Ecospace [10, 70]. Finally, the lack of noticeable herring fishery impacts on the three predators in our model which predominantly consume juvenile rather than adult herring (i.e., Pacific cod *Gadus macrocephalus*, lingcod *Ophiodon elongatus* and inshore rockfish *Sebastes* spp.) agrees with the conclusions of two recent studies [51, 52].

#### Management strategy parameter effects

Hockey-stick HCRs often noticeably outperformed their step and constant *F*_*target*_ counterparts (Figs 5 and 6) in terms of herring and predator biomasses (Fig 9), as previously found by the Lenfest Forage Fish Task Force [2]. However, the magnitude of their advantage varied with the similarity of the *F*_*target*_ and to a lesser extent, *B*_*lim*_ values. Furthermore, hockey-stick HCRs require exceptionally precise annual estimates of stock biomass and fishing mortality, which renders them a difficult and costly EBFM option to implement. A single-species MSE analysis [33] found no advantage in a hockey-stick HCR relative to the *BC* strategy. However, in this HCR, *B*_*lim*_ and *B*_*Ftarget*_ were set relative to *B*_*MSY*_ rather than *B*_*0*_, which has been found to reduce strategy performance [71]. Furthermore, the *B*_*lim*_ for this HCR was lower than in the *BC* strategy when rescaled relative to *B*_*0*_.

Reducing *F*_*target*_ thus appears to be the simplest approach to maintaining high herring and predatory mammal biomasses in the Northeast Pacific. This conclusion is consistent with the findings of the Lenfest Task Force [2], but not with those of Cleary et al. [33]. Simultaneously raising *B*_*lim*_ from 0.25*B*_*0*_ (current *BC* strategy) to 0.40*B*_*0*_ (*LF1* and *LF3*), as recommended by the Task Force [2], would enhance the effectiveness of *F*_*target*_ reductions. Raising *B*_*lim*_ to 0.70–0.80*B*_*0*_, also suggested by the Task Force [2], would likely be even more effective, but our EwE model could not consistently produce herring biomasses at this level, likely due to poor herring recovery or inadequate herring prey availability in the current Northeast Pacific ecosystem. A recent study [72] also recommended using *B*_*lim*_ > 0.25*B*_*0*_ in British Columbia herring fisheries to account for the ecosystem role of herring and for stock assessment error due to variable egg loss rates and spawn survey catchability.

Herring fishing at *F*_*MSY*_ caused stronger biomass changes in many functional groups than any other strategy tested (Fig 9). The resulting substantial reorganization of the food web was deleterious to herring predators and favourable to planktivores. Exploiting forage fish at *F*_*MSY*_ often has adverse effects on predators and food webs [2, 44] and carries the risk of forage fish stock depletion when assessment error combines with poor primary productivity [2, 15]. These findings apply equally to *MSY*_*s*_ and *MSY*_*e*_, although higher *B*_*lim*_ values somewhat mitigate their effects on functional group biomasses (Fig 9). However, unlike Walters et al. [44], we detected relatively strong negative effects on predator biomasses even when only one species (herring) was fished at *F*_*MSY*_. This suggests that *F*_*MSY*_ may be incompatible with herring EBFM, at least when not moderated by a precautionary hockey-stick HCR such as that applied to Norwegian spring-spawning herring [60]. Given that herring mortality in the postwar British Columbia reduction fishery often exceeded *F*_*MSY*_ [73, 74], this fishery likely had substantial adverse impacts on some herring predators.

It is noteworthy that the spawn-on-kelp fishery alone (*SOK* scenario), even assuming maximum incidental spawner mortality (*F*_*target*_ = 0.01), apparently has negligible effects on herring and predator biomasses (Fig 9). This finding agrees with that of Shelton et al. [29] based on single-species modelling and extends its applicability into the EBFM realm. Since most herring taken in the Northeast Pacific are pre-spawners fished for their roe, the *SOK* scenario may offer a more sustainable alternative to the roe herring fishery.

#### Policy tradeoffs

The EwE MSE results highlight a tradeoff between herring and predatory marine mammal biomasses on the one hand (ecological value) and herring catches and fisheries openings on the other (socioeconomic value). Many previous studies [1–3] have revealed such tradeoffs while also noting strong impacts of forage fish fisheries on seabirds and piscivorous fish. Unsurprisingly, grand mean herring catches were highest for those scenarios (constant *F*_*target*_ or step HCRs, high *F*_*target*_, low *B*_*lim*_) most strongly affecting herring and predatory mammal biomasses (Fig 12). The probability of herring fishery closure was highest for step HCRs with high *F*_*target*_ and *B*_*lim*_ values (Fig 14), suggesting potentially high socioeconomic costs for such management strategies. While mean catch was similar in the *BC* and *K* scenarios, as in the single-species simulations by Hall et al. [37], the probability of fishery closure in the *BC* scenario was approximately twice that observed in the single-species analysis. This is likely due to the interactive effects of stock assessment error and poor primary productivity discussed in detail below. It is worth noting that the *BC* strategy was introduced in 1986 to minimize the probability of herring fishery closure while maintaining stable but relatively high catches and spawning stock biomasses [33, 37].

Examining these tradeoffs with the Ecosim policy search routine, which combines ecosystem modelling with multi-attribute objective functions, offers a fruitful avenue for exploring precautionary, ecosystem-based and socioeconomically acceptable herring management strategies. This approach would allow researchers to model each strategy, incorporating the values and preferences of conservationists as well as commercial and aboriginal fishers, and thus search for a strategy that would best balance ecological and socioeconomic outcomes. However, given the substantial error accompanying *B*_*0*_, *B*_*lim*_ and *F*_*target*_ estimates obtained from stock assessment [14, 56], management decisions derived from HCRs based on these quantities are opaque to and thus often contested by stakeholders [56], e.g. Northeast Pacific aboriginal peoples reliant on herring [30–32].

Alternatively, ecosystem modelling and EwE MSE scenarios have been combined with practical ethics within an innovative, participatory value- and ecosystem-based management approach (VEBMA) to address the British Columbia herring fishery conflict [75]. Community and herring industry members were interviewed and asked to prioritize among a set of values and choose among fishery management scenarios and *B*_*lim*_ values for the herring fishery in Haida Gwaii. Local community members preferred scenarios and *B*_*lim*_ values modelled to yield the least ecosystem impacts, while industry members typically preferred the status quo [75, 76]. By making explicit the ecological, cultural and socioeconomic values at the science-policy interface, VEBMA offers an integrative, transparent deliberation and decision-support tool that exposes policy tradeoffs and highlights compromise solutions to facilitate collaborative governance among diverse stakeholders and affected local communities [75, 77].

#### Stock assessment error effects

In some runs of each EwE MSE scenario, simulated stock assessment error appears to have coincided with poor primary productivity years, exacerbating herring fishing effects on the food web (Fig 10). Overestimation of stock biomass by 30% (the maximum value permissible in our model settings) in a poor productivity year would create simultaneous top-down and bottom-up pressures on herring. This could lead to strong direct and indirect trophic effects on numerous functional groups. As primary productivity and stock assessment error were the only randomly varying factors in our simulations, an additive relationship between them is the most logical explanation for the strong herring fishing effects observed in the μ—0.675σ of group biomass distributions. For those functional groups (e.g. transient salmon and inshore rockfish) showing consistent negative effects across all strategies, poor primary productivity years alone appear to be responsible. Consecutive poor productivity years, accompanied by initially random but propagating stock biomass overestimations [56], could establish a pernicious positive feedback loop reducing many group biomasses. This interpretation agrees with recent findings [15, 16] regarding the vulnerability of forage fish to the combined effects of oceanographic fluctuations and fishing pressure, as well as with the conclusions of a single-species MSE analysis [33] on the sensitivity of the *BC* strategy performance to herring stock productivity. Therefore, accurate stock assessment and monitoring of oceanographic conditions are key to precautionary and ecosystem-based herring fisheries management.

#### Climate shift effects

The EwE MSE results suggest that the impacts of climate shifts on herring fisheries could be profound, as the performance of the *BC* management strategy relative to the current baseline (herring *F* = 0) is highly dependent on the primary productivity regime (Fig 11). While “no good productivity years” (*BC*_*0*.*75*_) caused several functional group biomasses to change more strongly than in the *BC* scenario, “only bad productivity years” (*BC*_*0*.*25*_) impacted most functional groups more strongly than *BC* or even *MSY*. Herring management strategy performance is therefore contingent on primary productivity levels and must be evaluated in this context. This is consistent with the bottom-up control of Northeast Pacific fisheries yields [78], although recent end-to-end modelling [61] indicates that this relationship may be complicated by plankton community dynamics, parameter uncertainty and representation of natural mortality. Our results also agree with recent projections of future climate change impacts on local aboriginal herring fisheries [79] and with contemporary findings [69] regarding the current primacy of climatic drivers over fishing pressure in affecting forage fish and predator biomasses in the California Current.

### Future research directions

Since the completion of these analyses, a new EwE plugin supplying improved MSE functionality, combined with extensive consideration of parameter uncertainty via Monte Carlo resampling and generation of multiple EwE models as platforms for MSE analysis, has been introduced [80] and applied to pressing issues in strategic management of North Sea fisheries [81]. This plugin, combined with an improved food web model [22], will allow us to explore the effects of model structure and resolution, parameter uncertainty and implementation error on the conclusions of the present study, as well as to simulate more complex HCRs [59] in a true MSE framework with full representation of all human and ecological subsystems [55].

## Conclusions

Ecopath, Ecosim and EwE MSE results show that Pacific herring is an important prey item for various Northeast Pacific predators, particularly marine mammals, and that its depletion could have notable cascading effects on predator populations and food web structure. EwE MSE simulations suggest that fishing strategies incorporating low *F*_*target*_, high *B*_*lim*_ and hockey-stick HCRs are particularly promising for precautionary and ecosystem-based herring fisheries management (corroborating the Lenfest Forage Fish Task Force findings), while unmoderated MSY strategies are incompatible with these approaches. Notably for British Columbia and Alaska, EwE MSE results also indicate that the SOK fishery traditionally practiced by coastal First Nations has minimal ecological impacts, while the existing British Columbia herring management strategy shows only modest impacts. However, fishing effects on herring and its predators may often be exacerbated by stock assessment error and poor primary productivity, raising the risk of strong ecosystem impacts under climate variability and shifts. The EwE MSE simulation results demonstrate that tradeoffs among the ecological, cultural, and socioeconomic values of Pacific herring must be weighed carefully, considering the impacts and risks to herring predators and the food web, as well as to commercial and aboriginal fisheries and local communities.

## Supporting information

S1 File Sensitivity analyses, supplementary references and figures. (DOCX)

S2 File Basic Ecopath parameters. (XLSX)

S3 File Basic Ecosim parameters. (XLSX)

S4 File Ecosim time series matrix. (XLSX)

S5 File Ecosim outputs. (XLSX)

S6 File MSE outputs. (XLSX)
