# Correction: Herring supports Northeast Pacific predators and fisheries: Insights from ecosystem modelling and management strategy evaluation

**DOI:** 10.1371/journal.pone.0207893

**Published:** 2018-11-16

**Authors:** Szymon Surma, Tony J. Pitcher, Rajeev Kumar, Divya Varkey, Evgeny A. Pakhomov, Mimi E. Lam

In the Author Contributions section, Mimi E. Lam (MEL) should be listed as one of the persons who worked on the methodology.
