# Correction: Herring supports Northeast Pacific predators and fisheries: Insights from ecosystem modelling and management strategy evaluation

**DOI:** 10.1371/journal.pone.0244032

**Published:** 2020-12-09

**Authors:** Szymon Surma, Tony J. Pitcher, Rajeev Kumar, Divya Varkey, Evgeny A. Pakhomov, Mimi E. Lam

The following information is missing from the Funding statement: This project has received funding (through an individual fellowship to MEL) from the European Union’s Horizon 2020 research and innovation programme under the Marie Skłodowska-Curie grant agreement No. 753937.
