# Supplementary material for: Herring supports Northeast Pacific predators and fisheries: Insights from ecosystem modelling and management strategy evaluation
Source: PLoS One. 2018 Jul 6;13(7):e0196307. doi: 10.1371/journal.pone.0196307 (PMC6034797; doi:10.1371/journal.pone.0196307)
Supplement: S1 File — (DOCX) [file pone.0196307.s001.docx]

**Herring supports Northeast Pacific predators and fisheries: insights from ecosystem modelling and management strategy evaluation**

*Szymon Surma, Tony J. Pitcher, Rajeev Kumar, Divya Varkey, Evgeny A. Pakhomov, and Mimi E. Lam*

Supporting information file S1: Sensitivity analyses, supplementary references and figures.

**S1.1 Sensitivity analyses**

*S1.1.1 Humpback whale diet*

We analyzed the sensitivity of the Ecosim and MSE results obtained for humpback whales to their diet composition in the Ecopath model, based on the trophic levels reported for these whales in the model area by Witteveen et al. (2011) from stable isotope analysis (3.5 for Northern BC and 3.4 for Southeast AK, no significant difference between regions). These values are substantially lower than that (3.65) derived from our original Ecopath model. The sensitivity analysis was based on two scenarios: (1) in which decreased humpback whale trophic level (3.4) was due in equal measure to reduced consumption of herring and other forage fish and (2) in which the same trophic level was obtained by reduced consumption of other forage fish only, relative to the diet composition in the original model (Figure S1.3.1).

In the Ecosim simulation (~95% herring depletion), the humpback whale diet in scenario 2 strongly decreased the magnitude of the change in humpback biomass, while that in scenario 1 reversed its sign from negative to positive (Figure S1.3.2). In the MSE simulations, there was no significant difference between the baseline and the most extreme fishing strategy (MSYe1) for either scenario 1 or 2, unlike in the original model. However, in scenario 1 this strategy was associated with a slight increase in humpback whale biomass, while in scenario 2 a slight decrease was observed (Figure S1.3.3).

These results suggest that current model predictions of humpback whale responses to herring fisheries are accompanied by substantial uncertainty. The trophic levels calculated by Witteveen et al. (2011) are themselves uncertain, however, due to their reliance on data from two years only and the assumptions implicit in the stable isotope analysis. Furthermore, as is the case for other baleen whales (Flinn et al. 2002), the proportions of prey categories (fish and euphausiids) in humpback whale diet vary on an interannual scale (Fleming et al. 2016). Finally, while regional variation in Northeast Pacific humpback whale trophic level detected by Witteveen et al. (2011) is matched by a similar pattern in fin whale diet (Mizroch et al. 2009), the average trophic level obtained by Witteveen et al. (2011) closely matches that used in our original model.

*S1.1.2. Seabird aggregation*

We further investigated the effects of herring fisheries on seabirds using an updated version of our model with scenario 2 diets for humpback whales and three separate seabird functional groups (piscivorous, teuthivorous and planktivorous) classified by diet (Kumar et al. 2016).

Piscivorous seabird biomass decreased by > 25% as a result of ~95% depletion of herring. Conversely, biomasses of teuthivorous and planktivorous seabirds increased by > 75% and > 40%, respectively. These changes were likely due to reduced competition from herring for zooplankton, resulting in increased squid biomass. In the MSE simulations, teuthivorous and planktivorous seabird biomasses were substantially higher under the most extreme fishing strategy (MSYe1) than in the baseline scenario. Piscivorous seabird biomass was lower under the most extreme strategy than in the baseline, but confidence intervals overlapped.

The results of this analysis suggest that herring fisheries effects on seabirds differ according to the latter’s dietary preferences and could in some cases be substantial. This finding agrees qualitatively with the conclusions of Cury et al. (2011), Koehn et al. (2016), Punt et al. (2016) and Sydeman et al. (2017) on the importance of adequate forage fish biomass to many seabird populations.

*S1.1.3. Vulnerability parameters*

We also examined the sensitivity of the Ecosim and MSE results to the Ecosim vulnerability parameters by replacing the parameter values used in the seabird analysis above with a set scaled to predator trophic level using an automatic routine in EwE 6.6 (developer version).

In the Ecosim simulation, biomass changes > 25% in response to ~95% herring depletion were seen in the same set of functional groups as in the original analysis (*F* = 1.7), except for seabirds, transient salmon and humpback, blue, fin and sperm whales (Figure S1.3.5).

Substantial responses were no longer seen in hake or humpback, blue, sei, and sperm whales. In turn, the responses of piscivorous seabirds (negative) and planktivorous seabirds, dogfish, salmon sharks, Pacific Ocean perch and sablefish (all positive) became noticeable.

However, the qualitative direction of changes in functional group biomasses under the most extreme herring fishing strategy remained the same, and changes in effect size due to the new parameters were 9% for adult herring and < 10% for ~95% of groups (i.e. all except juvenile herring, teuthivorous seabirds and planktivorous seabirds, for which the change in effect size likely also resulted from disaggregation of the original seabird group). These results suggest that the MSE simulation results are robust to uncertainty in vulnerability parameter values.

**S1.2. Supporting references**

Flinn, R.D., Trites, A.W., Gregr, E.J. and Perry, R.I. (2002) Diets of fin, sei, and sperm whales in British Columbia: an analysis of commercial whaling records 1963–1967. *Mar. Mamm. Sci.* 18: 663–679.

Fleming, A.H., Clark, C.T., Calambokidis, J. and Barlow, J. (2016) Humpback whale diets respond to variance in ocean climate and ecosystem conditions in the California Current. *Global Change Biol.* 22: 1214-1224.

Mizroch, S.A., Rice, D.W., Zwiefelhofer, D., Waite, J. and Perryman, W.L. (2009) Distribution and movements of fin whales in the North Pacific Ocean. *Mamm. Rev.* 39: 193–227.

Witteveen, B.H., Worthy, G.A.J., Wynne, K.M., Hirons, A.C., Andrews, A.G. III and Markel, R.W. (2011) Trophic Levels of North Pacific Humpback Whales (Megaptera novaeangliae) Through Analysis of Stable Isotopes: Implications on Prey and Resource Quality. *Aquat. Mamm.* 37(2): 101-110.

**S1.3. Supporting figures**

Figure S1.3.1. Proportions of herring, forage fish and euphausiids in three modelled humpback whale diets and in the total ecosystem biomass of humpback whale prey.

Figure S1.3.2. Changes in humpback whale biomass in response to ~95% herring depletion in Ecosim (sensitivity to modelled humpback whale trophic level and diet composition).

Figure S1.3.3. Humpback whale biomass densities observed in the baseline and most extreme MSE scenarios (sensitivity to modelled humpback whale trophic level and diet composition).

Figure S1.3.4. Grand mean functional group biomass changes for three seabird groups in response to ~95% herring depletion in Ecosim (sensitivity to seabird group aggregation).

Figure S1.3.5. Biomass densities of a) piscivorous, b) teuthivorous and c) planktivorous seabirds observed in the baseline and most extreme MSE scenarios (sensitivity to seabird group aggregation).

Figure S1.3.6. Grand mean functional group biomass changes exceeding 20% of baseline values from the Ecosim herring depletion simulation (vulnerability sensitivity analysis).

Figure S1.3.7. Humpback whale biomass densities across MSE scenarios. Error bars indicate ±2 SE.

Figure S1.3.8. Transient orca biomass densities across MSE scenarios. Error bars indicate ±2 SE.

Figure S1.3.9. Dolphin and porpoise biomass densities across MSE scenarios. Error bars indicate ±2 SE.

Figure S1.3.10. Seal biomass densities across MSE scenarios. Error bars indicate ±2 SE.

Figure S1.3.11. Adult herring biomass densities across MSE scenarios. Error bars indicate ±2 SE.

Figure S1.3.12. Juvenile herring biomass densities across MSE scenarios. Error bars indicate ±2 SE.

Figure S1.3.13. Hake biomass densities across MSE scenarios. Error bars indicate ±2 SE.

Figure S1.3.14. Blue whale biomass densities across MSE scenarios. Error bars indicate ±2 SE.

Figure S1.3.15. Fin whale biomass densities across MSE scenarios. Error bars indicate ±2 SE.

Figure S1.3.16. Sei whale biomass densities across MSE scenarios. Error bars indicate ±2 SE.

Figure S1.3.17. Sperm whale biomass densities across MSE scenarios. Error bars indicate ±2 SE.

Figure S1.3.18. Walleye pollock biomass densities across MSE scenarios. Error bars indicate ±2 SE.

Figure S1.3.19. Blue shark biomass densities across MSE scenarios. Error bars indicate ±2 SE.
